# The role of pharmacogenetics in the treatment of major depressive disorder: a critical review

**DOI:** 10.3389/fpsyt.2023.1307473

**Published:** 2023-11-10

**Authors:** Stefano Barlati, Alessandra Minelli, Gabriele Nibbio, Lorenzo Bertoni, Nicola Necchini, Stefano Paolini, Alessia Muscarella, Ughetta Bosco Ubertino, Irene Calzavara-Pinton, Antonio Vita, Massimo Gennarelli

**Affiliations:** ^1^Department of Clinical and Experimental Sciences, University of Brescia, Brescia, Italy; ^2^Department of Mental Health and Addiction Services, ASST Spedali Civili di Brescia, Brescia, Italy; ^3^Genetics Unit, IRCCS Istituto Centro San Giovanni di Dio Fatebenefratelli, Brescia, Italy; ^4^Department of Molecular and Translational Medicine, University of Brescia, Brescia, Italy

**Keywords:** antidepressant, effectiveness, major depressive disorder, personalized medicine, pharmacogenetic, remission, response, tolerability

## Abstract

Pharmacological therapy represents one of the essential approaches to treatment of Major Depressive Disorder (MDD). However, currently available antidepressant medications show high rates of first-level treatment non-response, and several attempts are often required to find an effective molecule for a specific patient in clinical practice. In this context, pharmacogenetic analyses could represent a valuable tool to identify appropriate pharmacological treatment quickly and more effectively. However, the usefulness and the practical effectiveness of pharmacogenetic testing currently remains an object of scientific debate. The present narrative and critical review focuses on exploring the available evidence supporting the usefulness of pharmacogenetic testing for the treatment of MDD in clinical practice, highlighting both the points of strength and the limitations of the available studies and of currently used tests. Future research directions and suggestions to improve the quality of available evidence, as well as consideration on the potential use of pharmacogenetic tests in everyday clinical practice are also presented.

## Introduction

Major Depressive Disorder (MDD) is a common psychiatric disorder, affecting more than 300 million people globally and representing an important cause of disability worldwide (1–3).

Symptomatic remission and maintenance of therapeutic effects over time are the primary goals of MDD treatment and the most common first-line therapeutic strategy for moderate to severe MDD is pharmacological: although different classes of antidepressants are currently available, the pharmacological approach to this disorder is commonly a process of trial and error, thus, it may (and often does) take several attempts to identify the optimal treatment for an individual patient (2–4).

Approximately one third of patients achieve remission after the first therapeutic trial, whereas about another third develop a treatment-resistant form of depression (2, 5, 6).

The inherent biological and environmental heterogeneity among patients with this disorder could be one of the causes of the high non-response and incomplete remission rates, suggesting the potential usefulness of identifying specific biomarkers able to predict the response to antidepressants and allowing to individually tailor the treatment for each subject (2, 7–9).

Specific guidance for clinicians to navigate which antidepressant is better suited for each patient, therefore, is much needed: pharmacogenetics aims at doing precisely that by combining aspects such as genetic variability, pharmacokinetics, and clinical outcomes, allowing drug selection based on the genomic characteristics of the individual patient (10).

In the context of MDD, current evidence focuses on pharmacokinetics, and in particular on the variability of two genes involved in drug metabolism: CYP2C19 and CYP2D6 (10–14).

Other genes have also been identified as biomarkers in the response to antidepressant therapy (e.g., some serotonin’s receptors and pathway’s molecules like SCL6A4 and HTR2A) and, despite more limited clinical validity and efficacy, have also been included in many commercially available pharmacogenetic test panels (3, 10, 15–22).

Although several randomized controlled trials (RCTs) have yielded interesting results regarding the impact of pharmacogenetic tests on the efficacy outcome of MDD patients’ treatment, conflicting opinions remain regarding their usefulness in everyday clinical practice (2–4, 23–25).

The aim of the present narrative and critical review is to analyse the available literature in order to assess, without neglecting possible criticalities and limitations, the evidence on the efficacy, safety and applicability of currently available pharmacogenetic tests in the context of MDD treatment.

## Currently available evidence

A review conducted in 2022 (4) with the aim of providing an assessment of the clinical methodological characteristics of RCTs that used pharmacogenetic testing in the treatment of patients with MDD identified seven multicenter prospective RCTs (26–32): many of the analyzed RCTs assessed as a primary outcome the improvement at 8 weeks of depressive symptomatology through different scales administered by clinicians (27–30, 32). Moreover, secondary and tertiary outcomes were the response and remission rates at different times, the changes in depressive symptom scores, as well as response and remission rates at different times assessed with a self-administered scale, the changes in MDD-related symptom scores, such as anxiety, and side effects.

Also in 2022, Brown et al. published a systematic review evaluating 10 RCTs and 3 open-label trials that analyzed the effectiveness of pharmacogenetic tests in guiding the choice of antidepressant medication: on the basis of these RCTs, a meta-analysis was then conducted with the aim of establishing whether the use of pharmacogenetics is associated with a higher rate of remission of depressive symptoms and whether the results obtained in this respect are comparable to those found in two previous meta-analyses (10, 23, 33). In this study, a total of 4767 patients were enrolled among all the trials involved and the majority (9/13) of the analyzed trials assessed their primary outcome 8 weeks after baseline (range 8–24 weeks). Furthermore, although the genes considered for pharmacogenetic testing were different in the various studies, CYP2C19 and CYP2D6 were always included in the analyses (10).

In 2023 Wang et al. conducted a systematic review of 1779 articles in the literature and published a meta-analysis from 11 included RCTs and a total of 5347 patients. The primary outcome of this study was to determine the rate of response (≥ 50% reduction on the Hamilton Depression Rating Scale -HAM-D or on the Patient Health Questionnaire -PHQ-9 scale score compared to baseline) and remission (HAM-D score ≤ 7 or ≤ 5 on the PHQ-9 or ≤ 2 on the Clinician Global Impression -CGI) at 4, 8, 12, and 24 weeks between patients in the TAU group and subjects undergoing pharmacogenetic guided antidepressant treatment (PGx). Another outcome considered was medication congruence in 30 days (participants were prescribed antidepressant medication that was classified as having no drug-gene interaction or moderate drug-gene interaction) (3).

One of the largest available individual studies is that conducted by Oslin et al. (34), a pragmatic single-blind trial conducted in 22 Department of Veterans Affairs (VA) medical centers including 1944 participants randomized to receive antidepressant treatment based on pharmacogenetic test results (PGx group) or according to standard clinical practice, with the results of pharmacogenetic test being disclosed 24 weeks later (TAU group).

## Improvement of depressive symptoms, response, and remission rates

According to Minelli et al. (4), just one work (28) reported that patients assigned to the pharmacogenetic group show a greater improvement in depressive symptoms severity considered as a continuous dimension (mean change in the HAM-D total score) at 8 weeks after the start of treatment, while no substantial differences were observed in the other included studies.

However, regarding response and/or remission rates, several RCTs have shown significant results in favor of the PGx-guided group of patients and, in particular, one study found that, at 12 weeks, the response rate in patients diagnosed with severe MDD was higher in the PGx-guided group than in the TAU group (29). In addition, also for patients in the PGx-guided group who had one to three previous antidepressant drug treatments in their history, a minimal benefit was shown compared to TAU. Regarding response rate, this study found significant differences in favor of the PGx-guided group at 12 weeks (measured with the Patient Global Impression of Improvement -PGI-I scale) and at 6 and 12 weeks (measured with the HAM-D scale). For drug-naive subjects and those who had received four or more treatments in the past, on the other hand, no significant improvements were shown.

Additionally, in the 2018 study by Bradley et al., *post hoc* analyses stratified by severity of the current depressive episode were performed. It was found that at both 8 and 12 weeks, response and remission rates in patients diagnosed with severe MDD were higher in the PGx-guided group than in the TAU group. In contrast, no significant improvements were found in patients diagnosed with mild MDD (26). Similarly, the study conducted by Greden et al. the improvement of symptoms in patients in the PGx-guided group continued until week 24, with an almost double remission rate from week 8 to week 24. Thus, according to the authors, pharmacogenetic tests can guarantee a lasting effect of antidepressant drugs. However, an important limitation emerged: only patients in the PGx-guided group were observed over a long-time interval, without comparison with those in the TAU group (27).

These results are in line with those of Brown et al. (10), where the pooled risk ratios of these studies suggest that pharmacogenetic tests result in a modest but significantly favorable effect on the remission of depressive symptoms. This effect is significantly increased in subjects with a diagnosis of moderate to severe MDD and a history of non-response or side effects during antidepressant treatment. Subjects who received PGx-guided medications had a 41% higher chance of obtaining symptom remission compared to patients in the TAU group (10). Notably, this effect was found to be lower than that reported in the two previous meta-analyses, from which it was found that PGx-guided antidepressant treatment increased the probability of achieving remission of depressive symptoms by 71% and 74% (23, 33).

In the 2023 meta-analysis by Wang et al. the response rates and the remission rates for patients in the PGx-guided group were significantly higher than those in the TAU group at two timepoints (8 and 12 weeks). However, no significant between-groups differences were observed for the response rates and the remission rates at 4 weeks and the 24 weeks timepoints (3).

In the 2022 study by Oslin et al. (34) a significant group effect was found with a higher remission rate in the PGx driven in several timepoints: significant between-groups differences were observed at the at the 8 and the 12 weeks assessments. However, no significant effect was reported at 4, 8, and 24 weeks: in fact, remission criteria were met by 130 participants in the PGx group and by 126 in the TAU group at the endpoint assessment. Secondary outcomes of treatment response and reduction in symptom severity were also favorable for patients in the PGx-guided group compared to the TAU group (34).

In conclusion, there were small overall positive effects over 24 weeks, with peak differences at the start of the study and no significant differences in remission at 24 weeks. The secondary outcomes of response and symptom reduction analyzed also followed a similar pattern.

## Prescription of medications, tolerability and side effects

Two studies (27, 32) reported an increase in the prescribing of congruent medication by physicians for patients in the PGx-guided group but not for those in the TAU group. In two other studies (28, 29) greater tolerability and fewer side effects (as measured by the Frequency, Intensity and Burden of Side Effects Ratings scale) emerged in patients in the PGx-guided group compared to those in the TAU group (4).

In Wang et al. (3), on the other hand, a significant reduction in drug congruence at 30 days was shown in the group of patients in the PGx group compared to those in the TAU group.

Moreover, Oslin et al. (34) found that among patients who received an antidepressant drug, those in the guided PGx group were more likely to receive one without a potential drug-gene interaction. Specifically, the estimated risks of being treated with a medication with no, moderate and substantial drug-gene interaction for the PGx group were of 59.3, 30.0, and 10.7%, respectively. Conversely, the rates were of 25.7, 54.6, and 19.7% for the TAU group. Therefore, participants in the PGx-guided group were less likely to receive medications with moderate or substantial drug-gene interactions.

## Discussion

Currently available evidence highlighted a number of advantages related to the use of pharmacogenetic tests in the treatment of MDD.

First, most of the studies quantified a significant improvement in response and remission rates in subjects undergoing PGx-guided antidepressant treatment compared to patients in the TAU group.

Increasing the efficacy and accuracy in the choice of antidepressant treatment represents a major challenge as non-response or the development of side effects to a given drug may lead to discontinuation and non-adherence to therapy, resulting in increased social and health care costs due to the development of treatment-resistant depression or due to increased risk of relapse (4, 10, 35).

Specific clinical phenotypes, such as melancholic or anxious depression, may predict a differential response to antidepressants (36). As MDD is not a uniform disorder but includes heterogeneous clinical conditions, which may be related to specific biological imbalances that can induce different response patterns to certain classes of antidepressants, pharmacogenetic may be a tool to demonstrate the biological basis of these differences (37).

Although available studies show promising results, they present several critical issues that deserve consideration. Although the PGx-guided group showed better response and remission rates, uneven time points for outcome assessment were taken into account in most of the studies conducted (4). In this regard, it is important to analyse clinical outcomes after 8 weeks, which represent the usual duration of treatment of a depressive episode in the acute phase, and to prolong the evaluation for 12 or 24 weeks, as significant changes of clinical relevance could occur during this period of time and the duration of response and sustained remission represents aspects to that should be carefully evaluated (4, 38). For example, in the work by Wang et al. (3) a significant increase in response and remission rates was found in PGx-guided subjects compared to the TAU group at 8 and 12 weeks, whereas no significant difference was observed between the two groups at 4 and 24 weeks. The reasons for this lack of difference could be explained by the long onset time of the antidepressant drugs for the 4-week assessment, whereas for the 24-week assessment the pharmacogenetic tests may have favored the acceleration of the process of exclusion of ineffective drugs, resulting in an improved therapeutic efficacy of drugs, according to a “catalyst-like” effect (3). Another aspect to be emphasized is that, in some studies, the lack of prescriber and/or rater blindness does not make it possible to exclude a possible influence on the assessment of outcomes (there could be a significant risk of performance and detection bias) (33, 39).

Another potential issue regards inclusion and exclusion criteria. Future studies should consider a more homogeneous approach to the inclusion of participant and should consider more carefully some important aspects defining the clinical characterization of MDD. In particular, clinical subtypes defined by the severity of depressive symptoms in specific domains, such as melancholic characteristics, anxiety and psychotic symptoms, atypical and mixed features, as well as other elements such as seasonality of the episodes, suicidality, the overall clinical staging of the disorder, personality traits and potential personality disorders of participants should be considered and accounted for. Another important elements are comorbidity, including psychiatric issues beside affective and personality disorders, alcohol and substance abuse, medical comorbidities, history of trauma, and family history of mental disorders (4, 40).

Some studies included subjects in need of *de novo* antidepressant treatment (26, 29, 31), while others included participants that were already on antidepressant medication and required a drug switch due to lack substantial positive effects or discontinuation of treatment due to reported occurrence of adverse events (27, 28, 30, 32). This mode of recruitment does not provide clarity, in terms of finding a good cost-benefit trade-off, as to whether pharmacogenetic testing should be recommended to patients before or after initial treatment ineffectiveness (preventive or reactive testing) (4, 41, 42).

Regarding demographic characteristics, most of the studies included individuals of Caucasian origin, an aspect that severely limits the generalizability of the results (2, 4, 39).

Beside potential methodological issues, several studies are industry-funded or present substantial financial conflicts of interests (4, 41). Often each institution provides different testing tools and services than the other companies and most commercially available tests contain more data/genes than FDA-approved tests or by tests reviewed in available guidelines. These aspects could call into question the complete reliability of the results generated by these tools (3, 41).

Although multiple articles have highlighted that pharmacogenetic tests have the potential to ensure cost reduction with their use (43–45), their true cost-effectiveness remains unclear, highlighting the need for further studies to be conducted in this regard (3, 46).

Also, the role of pharmacogenetic on side effects also remains a point to be investigated more thoroughly, considering that in many of the studies considered, no structured and validated tools were used to ensure a thorough analysis (4, 10).

Finally, the use of self-report scales for depressive symptoms and other clinical outcomes was not implemented in most of the analyzed studies, and therefore the assessment of the participants’ own perspective is currently lacking: this is a significant issue, as the patients’ perspective is of substantial importance as is closely related to real-world functional remission of the disorder (4, 47).

In conclusion, interesting insights have emerged in this review, suggesting that pharmacogenetic tests may prove to be an effective strategy in the treatment of MDD patients; however, their applicability in daily clinical practice is still a gray area that is difficult to clarify. In order to be able to reduce these doubts, multicenter double-blinded studies on larger and demographically more heterogeneous samples are currently needed: these studies could potentially guarantee the generalizability of the results, and would allow to more accurately assess the long-term efficacy, cost-effectiveness, tolerability and safety of this therapeutic strategy in the choice of antidepressant drug. In addition to these more technical aspects, it will be necessary in the future to adequately educate clinicians about the characteristics and methods of using pharmacogenetic tests. In the light of the promising results shown in this review, the previously highlighted limitations must represent a starting point from which to begin bridging the knowledge gap that makes their use in clinical practice uncertain and whose overcoming could constitute a marked direction change toward a better treatment pathway for MDD patients.

## Author contributions

SB: Conceptualization, Funding acquisition, Resources, Supervision, Validation, Writing−review and editing. AM: Conceptualization, Funding acquisition, Resources, Supervision, Validation, Writing−review and editing. GN: Conceptualization, Supervision, Writing−original draft, Writing−review and editing. LB: Data curation, Formal analysis, Investigation, Writing−original draft. NN: Data curation, Formal analysis, Investigation, Writing−original draft. SP: Data curation, Formal analysis, Investigation, Writing−original draft. AM: Investigation, Writing−original draft. UBU: Investigation, Writing−original draft. IC-P: Investigation, Supervision, Writing−original draft, Writing−review and editing. AV: Conceptualization, Funding acquisition, Resources, Supervision, Validation, Writing−review and editing. MG: Conceptualization, Funding acquisition, Validation, Writing−review and editing.
